# Multimodal imaging of a sporadic retinal astrocytic hamartoma simulating retinoblastoma in a newborn

**DOI:** 10.3205/oc000198

**Published:** 2022-05-20

**Authors:** Bilge Batu Oto, Aslihan Yilmaz Çebi, Oguzhan Kiliçarslan, Ahmet Murat Sarici

**Affiliations:** 1 Ophthalmology Department, Istanbul University-Cerrahpasa, Cerrahpasa Faculty of Medicine, Istanbul, Turkey

**Keywords:** retinoblastoma, retinal astrocytic hamartoma, neoplasms, optical coherence tomography, retinal tumors

## Abstract

**Introduction:**

To report a sporadic astrocytic hamartoma simulating retinoblastoma in a newborn.

**Methods:**

Clinical data was reviewed retrospectively.

**Results:**

A 3-month-old baby with a history of perinatal asphyxia was referred to our ocular oncology clinic with suspected retinoblastoma in the left eye. Dilated fundoscopy revealed a solitary tumor covering the optic disc at the left eye. The whitish-yellow lesion was well-defined, opaque, and minimally calcified. High internal reflectivity and posterior shadowing due to the intralesional calcification, and intratumoral cystic spaces were observed in B-scan ultrasound imaging. Optical coherence tomography imaging showed an intraretinal tumor with cystic spaces and posterior shadowing. The tumor was diagnosed as an astrocytic hamartoma. The systemic evaluation was negative for phacomatoses. The lesion has been observed with multimodal imaging for six years without significant changes.

**Conclusions:**

Retinal astrocytic hamartomas are benign tumors that arise within the retinal nerve fiber layer. Differential diagnosis constitutes high importance since they may be misdiagnosed as retinoblastoma, and therefore may be overtreated. Whereas retinoblastoma requires immediate treatment, retinal astrocytic hamartomas are commonly followed-up. Multimodal imaging with B-scan ultrasonography and optical coherence tomography are useful in distinguishing those two entities.

## Introduction

Retinal astrocytic hamartomas (RAHs) are benign, glial tumors that arise within the retinal nerve fiber layer. They can occur as isolated sporadic lesions or may be associated with genetic neurocutaneous syndromes such as neurofibromatosis or tuberous sclerosis [1]. The fundoscopic finding is usually a well-demarcated, multilobulated, whitish-yellow lesion that can be located in the posterior pole or peripheral retina. Sporadic cases tend to be unilateral and solitary [2]. Retinoblastoma (RB) is the most common intraocular malignancy in children. Its early diagnosis and accurate treatment are critical. However, due to the benign nature of RAHs, periodical observation can be enough for their management. Herein, we review a case of sporadic retinal astrocytic hamartoma mimicking retinoblastoma, which remains stable during the five-year follow-up period.

## Case description

A 3-month-old female patient was referred to our ocular oncology clinic with suspected retinoblastoma in the left eye. She was born at 36 weeks of gestation because of increased perinatal asphyxia risk due to a nuchal cord. She was incubated and had been treated with oxygen for fifteen days. The tumor had been detected at the retinopathy of prematurity screening visit.

**Figure 1:**
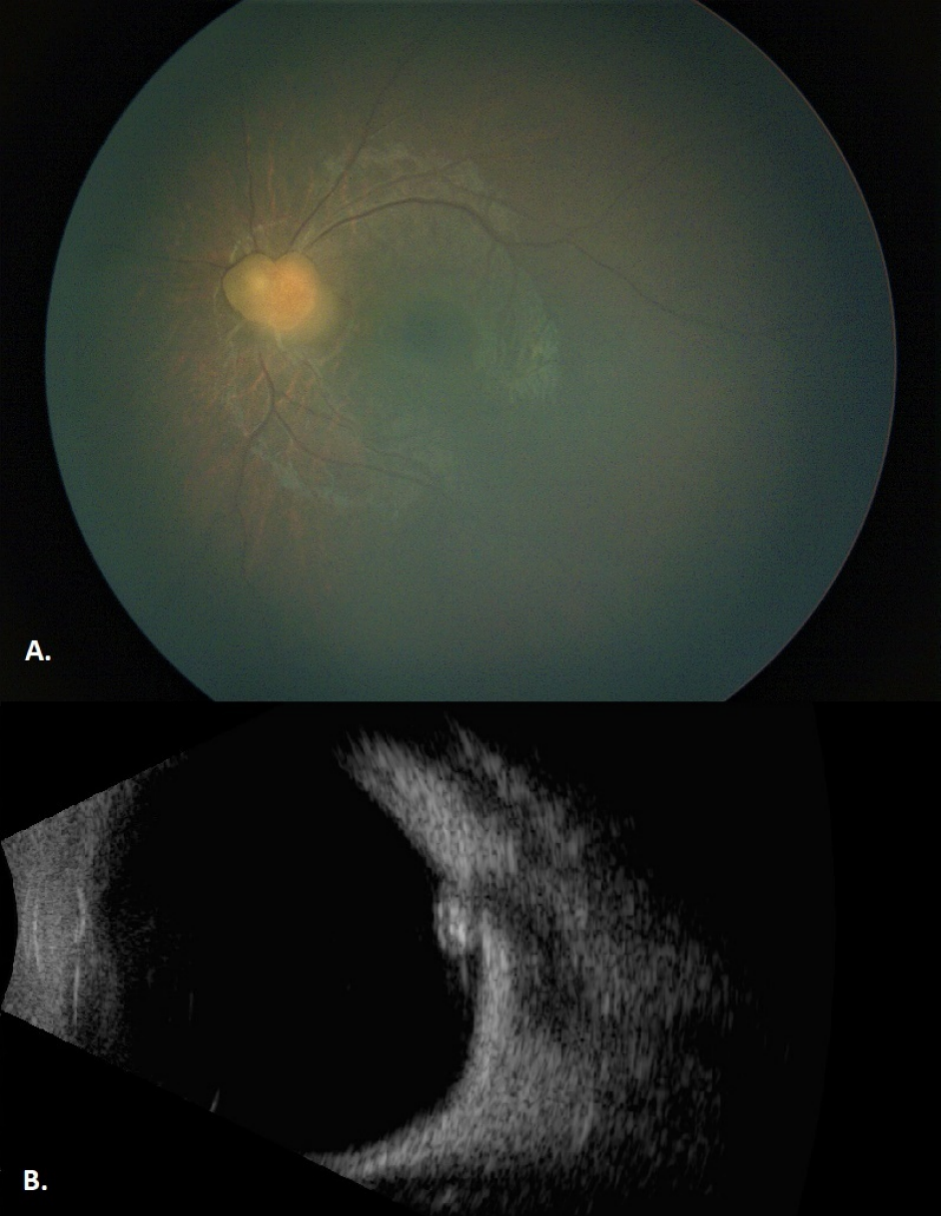
A) Fundus image of the left eye at initial examination shows a well-defined, opaque, minimally calcified, whitish-yellow lesion that is covering the optic disc. B) B-scan ultrasonography image of the left eye at initial examination shows high internal reflectivity, posterior shadowing, and intratumoral cystic spaces.

In our first examination, the patient’s vision was central, steady, maintained (CSM) at the right eye (OD), and uncentral, unsteady, unmaintained (UCUSUM) at the left eye (OS). There was a relative afferent pupillary defect at the left eye. Slit-lamp biomicroscopy and intraocular pressure measurement were normal in both eyes. Dilated fundoscopy was normal at the right eye, but revealed a solitary tumor covering the optic disc at the left eye. It was a well-defined, opaque, minimally calcified, whitish-yellow lesion. Distances from the fovea and the optic disc were 3 mm and 0 mm, respectively. On B-scan ultrasonography, the dimensions were 3x3x2 mm. High internal reflectivity and posterior shadowing due to the intralesional calcification, and intratumoral cystic spaces were noted (Figure 1 (Fig. 1)). There were no vitreous seedings, feeding vessels, or extraretinal tumor extension. The tumor was diagnosed as an astrocytic hamartoma. Although the medical history was negative for seizures or skin lesions, the patient was referred to a pediatrician and systemic diseases were excluded.

Observation was recommended for the management. Regular comprehensive eye examination was applied for six years with six-month intervals. At each visit, binocular indirect ophthalmoscopy, fundus photography, and B-scan ultrasonography were performed. Biomicroscopy was bilaterally normal. Fundoscopic findings or ultrasonography measurements did not significantly change throughout the six-year follow-up period. Optical coherence tomography scans showed the intratumoral cystic spaces as well, with posterior shadowing due to the intralesional calcification. The final vision was 20/20 OD and hand movement OS. Likewise, periodical screening had not revealed any systemic involvement.

## Discussion

Retinal astrocytic hamartomas (RAHs) are benign tumors that arise from glial astrocytes. They usually present as well-demarcated, mulberry-like lesions with intratumoral calcification [3]. When the fovea and the optic disc are preserved, they typically have an asymptomatic, indolent course of the disease. Complications such as retinal exudates and hemorrhages, cystoid macular edema, vitreous hemorrhage, retinal detachment, and neovascular glaucoma rarely occur. RAHs may be associated with systemic phakomatose syndromes like neurofibromatosis and tuberous sclerosis. They are the most common ocular finding of tuberous sclerosis [2]. Phakomatose-related RAHs tend to be bilateral, multiple, and they have a more aggressive tumor growth with an increased risk of complications [4]. In our case, it was a sporadic, unilateral, solitary tumor. We did not observe any tumor-related complications during the follow-up period. The systemic evaluation did not reveal any diseases.

Differential diagnosis of RAH includes retinoblastoma, choroidal osteoma, chorioretinitis, amelanotic melanoma, and von Hippel angiomas [4]. Drewe et al. [5] reported misdiagnosis of RAH as RB in 1985. Regarding the available treatment options, this misdiagnosis resulted in enucleation of the eye in that period [5]. Although the current recommended treatment for RBs that have no vitreous seeding or extraretinal extension is globe-salvaging therapies such as selective intraarterial chemotherapy [6], the importance of accurate and timely differential diagnosis of RB cannot be adequately emphasized. In our case, intralesional calcification and the absence of a characteristic mulberry-like appearance led to suspicion of RB. Well-defined margins, absence of feeding vessels or vitreous seeding, and presence of intratumoral cystic spaces were important for differential diagnosis.

**Figure 2:**
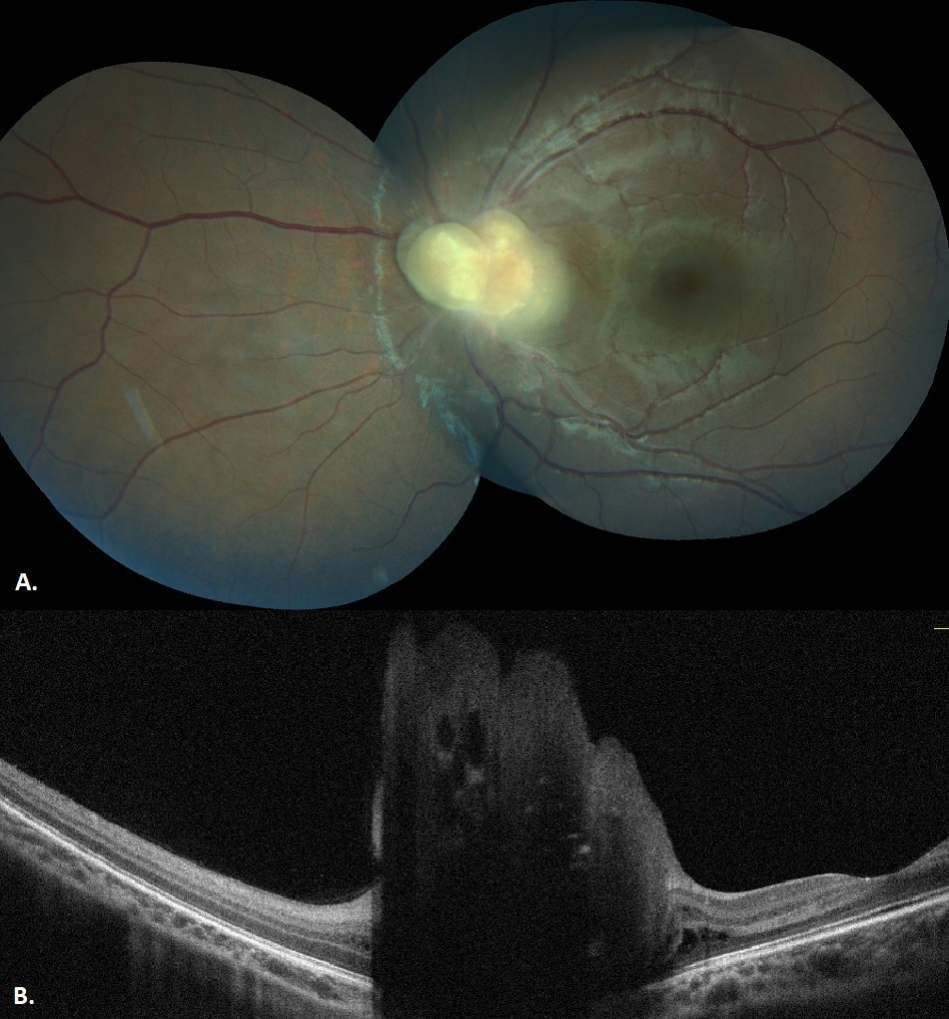
A) Fundus image of the left eye after a six-year follow-up shows a minimal change. B) Optical coherence tomography image of the left eye after six-year follow-up shows intratumoral cystic spaces and acoustic shadowing due to the calcification.

Unilateral, solitary RAHs are risky of misdiagnosis. Multimodal imaging of tumors can be helpful in identifying the true pathology. In recent years, optical coherence tomography (OCT) technology proved that RAH is confined to the retinal nerve fiber layer (RNFL), and the outer retinal segments are anatomically normal. There may be intratumoral empty spaces, posterior shadowing, and vitreoretinal traction [2]. In an observational study from Welch et al. [7], 16 eyes with retinoblastoma were evaluated with OCT after a session of chemotherapy. The tumor origin was found to be either in the inner nuclear layer or in both of the inner and outer nuclear layers [7]. Discontinuity of the inner nuclear, outer plexiform, outer nuclear layers, and the external limiting membrane was detected in all patients [7]. In our patient, OCT images showed that the tumor mainly involved the inner retinal layers, including the retinal nerve fiber layer. Intratumoral empty spaces and acoustic shadowing due to the intratumoral calcification were observed (Figure 2 (Fig. 2)). This moth-eaten appearance was defined as type 3 RAH by Pichi et al. and found to be correlated with the presence of subependymal giant cell astrocytomas [8].

## Conclusion

To summarize, a six-year follow-up without any change confirms the benign nature of retinal astrocytic hamartomas. Differential diagnosis from retinoblastoma is of utmost importance. Otherwise, periodical follow-up is sufficient for their management. Our patient is young and may yet develop the signs of a phakomatosis. The child should be referred to a pediatrician for systemic screening and follow-up.

## Notes

### Authors’ ORCIDs

- Bilge Batu Oto: 0000-0002-9729-2577
- Aslihan Yilmaz Çebi: 0000-0001-7919-9099
- Oguzhan Kiliçarslan: 0000-0003-4061-2047
- Ahmet Murat Sarici: 0000-0002-9061-4385

### Competing interests

The authors declare that they have no competing interests.
